# Biological effects of sub-lethal doses of glyphosate and AMPA on cardiac myoblasts

**DOI:** 10.3389/fphys.2023.1165868

**Published:** 2023-04-24

**Authors:** Elisa Arrigo, Sara Gilardi, Luisa Muratori, Stefania Raimondo, Daniele Mancardi

**Affiliations:** ^1^ Department of Clinical and Biological Sciences, University of Torino, Turin, Italy; ^2^ Neuroscience Institute Cavalieri Ottolenghi (NICO), University of Torino, Turin, Italy

**Keywords:** AMPA, ROS, cardiac myoblasts, mitochodria, glyphosate, H9c2

## Abstract

**Introduction:** Glyphosate is the active compound of different non-selective herbicides, being the most used agriculture pesticide worldwide. Glyphosate and AMPA (one of its main metabolites) are common pollutants of water, soil, and food sources such as crops. They can be detected in biological samples from both exposed workers and general population. Despite glyphosate acts as inhibitor of the shikimate pathway, present only in plants and some microorganisms, its safety in mammals is still debated. Acute glyphosate intoxications are correlated to cardiovascular/neuronal damages, but little is known about the effects of the chronic exposure.

**Methods:** We evaluated the direct biological effects of different concentrations of pure glyphosate/AMPA on a rat-derived cell line of cardiomyoblasts (H9c2) in acute (1–2 h) or sub-chronic (24–48 h) settings. We analyzed cell viability/morphology, ROS production and mitochondrial dynamics.

**Results:** Acute exposure to high doses (above 10 mM) of glyphosate and AMPA triggers immediate cytotoxic effects: reduction in cell viability, increased ROS production, morphological alterations and mitochondrial function. When exposed to lower glyphosate concentrations (1 μM—1 mM), H9c2 cells showed only a slight variation in cell viability and ROS production, while mitochondrial dynamic was unvaried. Moreover, the phenotype was completely restored after 48 h of treatment. Surprisingly, the sub-chronic (48 h) treatment with low concentrations (1 μM—1 mM) of AMPA led to a late cytotoxic response, reflected in a reduction in H9c2 viability.

**Conclusion:** The comprehension of the extent of human exposure to these molecules remains pivotal to have a better critical view of the available data.

## 1 Introduction

Glyphosate [IUPAC name N-(phosphonomethyl) glycine] is a synthetic phosphonic amino derivative of glycine, which disrupts the shikimate pathway by inhibiting the activity of 5-enolpyruvylshikimate-3-phosphatase (EPSP) synthase. This metabolic pathway is used by plants and several microorganisms for the biosynthesis of folate and aromatic aminoacids (Bai and Ogbourne, 2016). Glyphosate (Gly) is the active compound of a large part of non-selective herbicidal (glyphosate-based herbicidal, GBHs), being the most used worldwide since middle 70s (Torretta et al., 2018).

Gly is absorbed through leaves and stems and it is transported from roots to edible parts (Tong et al., 2017). In agriculture, genetically modified Gly-resistant crops (as soybean, cotton, corn, etc.) are extensively used and, because of their resistance they accumulate Gly at high concentrations (Xu et al., 2019). Once applied, Gly undergoes degradation mainly by a process known as mineralization, which leads to different byproducts, with aminomethyl phosphonic acid (AMPA) as the main metabolite. The kinetic of this mechanism is highly dependent on soil pH and minerals concentration. Other processes that determine Gly fate are immobilization and leaching: the first one leads to soil adsorption/accumulation, while the second results in water contamination (Bai and Ogbourne, 2016). Gly and AMPA are highly soluble in water and their persistence is variable depending on water conditions with half-lives ranging from few days to several weeks (Tomlin, 2009; Grandcoin et al., 2017; ATSDR, 2020; Goncalyes et al., 2020). In soil, Gly and AMPA accumulate with a discrete persistence with half-lives depending on factors such as pH, salinity, microbial composition, spanning from few days up to about a year (Bai and Ogbourne, 2016; Bento et al., 2016; Domínguez et al., 2016; Grandcoin et al., 2017; ATSDR, 2020).

Given the massive use of GBHs, Gly and AMPA are frequently detected in different water and food samples and classified as pollutants (Bai and Ogbourne, 2016; Bonansea et al., 2017; Silva et al., 2018; Xu et al., 2019; Okada et al., 2020; Marques et al., 2021; Pelosi et al., 2022). The constant presence represents not only an ecological burden but also a potential indirect threat to both animal and human health. Gly and AMPA were, in fact, found in urines of both occupationally or para-occupationally exposed workers (from 0.26 to 73.5 μg/L) and in general population (from 0.16 to 7.6 μg/L) (Krüger et al., 2014; Niemann et al., 2015; Gillezeau et al., 2019; Perry et al., 2019; Mesnage et al., 2022a). Indeed, this type of report suffers from inconstant technical approaches that fail to allow a reliable comparison, mostly because the available studies are based on very different methodologies for Gly and AMPA quantification (gas chromatography, liquid chromatography or ELISA) (Valle et al., 2019). Liquid chromatography is the elective analytical technique for glyphosate determination because of its flexibility and availability in different types of laboratories. This technique can be coupled with different detector types (i.e., ultraviolet-visible, fluorescence, mass spectrometry, etc.) many of which are applicable to Gly quantification. Every technique needs various degrees of technical skills to be performed and requires substantially different investments, and each of them can reach different levels of sensitivity (Moldovan et al., 2023). Hence, more accurate and standardized procedures are needed to reliable and repeatable measurements of Gly and AMPA concentration in biological samples and, therefore, an accurate evaluation of exposure extent.

Despite its selective mechanism of action, Gly has been proven to have either acute or chronic toxicity in different off-target non-mammals animal species, such as amphibians, annelids, arthropods, fishes and birds (Antón et al., 1994; Contardo-Jara et al., 2009; Roy et al., 2016; Gill et al., 2018; Jin et al., 2018). However, these effects were more severe when animals were exposed to Gly formulation than the molecule alone, suggesting that the adjuvants (such as surfactants) act in synergy, amplifying the toxicity.

As of today, the safety of Gly in mammals is still under debate. Acute intoxications due to GBHs ingestion are reported to strongly affect cardiovascular system (Bradberry et al., 2004; Gress et al., 2015; Brunetti et al., 2020; Hu et al., 2021), as well as to cause gastrointestinal and respiratory symptoms, hypotension and consciousness alteration (Lee et al., 2000; Bradberry et al., 2004); however, these effects are due to very high levels of Gly and adjuvants and are in line with accidental intake and does not reflect the low, although daily, exposure of the general population. The long-term effects of a chronic exposure to Gly and AMPA are not clear. Some *in vitro* studies on different mammalian cell lines showed Gly (or its formulations) to be genotoxic (Benachour and Séralini, 2009; Martini et al., 2012; Mesnage et al., 2013; Townsend et al., 2017; Santovito et al., 2018; Mesnage et al., 2022b), cytotoxic (Townsend et al., 2017; Vanlaeys et al., 2018; Hao et al., 2020; Martínez et al., 2020) and reprotoxic (Gasnier et al., 2009; Clair et al., 2012; De Liz Oliviera Cavalli et al., 2013; Anifandis et al., 2017; Stur et al., 2019; Hao et al., 2020; Jarrell et al., 2020; Cao et al., 2021; Mohammadi et al., 2022). Gly toxicity is usually associated with oxidative stress, dysfunctional mitochondria dynamics and bioenergetics. The sensibility to Gly seems to be cell specific; only few studies demonstrated Gly toxicity in concentrations below the human Acceptable Daily Intake (1.0 mg/kg) (Santovito et al., 2018) and not related to the adjuvants present in its formulations.

In the present work, we evaluate the direct biological effects of different concentrations of pure Gly or AMPA on a rat-derived immortalized cell line of cardiomyoblasts (H9c2), recognized as a valuable tool for investigating *in vitro* effect of toxic factors on myocardial and muscle-skeletal immortalized cells (Branco et al., 2015; Bouleftour et al., 2021; Onódi et al., 2022).

In the first part of the study, we simulated an acute exposure to high levels (10–20 mM) of Gly or AMPA. We eventually shifted to lower concentrations (1 μM—1 mM) in order to identify a sub-lethal range to mimic the biological effects of acute and sub-chronic treatments. We evaluated changes in cell viability, morphology, ROS production and mitochondrial distribution and mass.

## 2 Materials and methods

### 2.1 Solutions & reagents

MTT (3-(4,5-dimethylthiazol-2-yl)-2,5-diphenyl-2H-tetrazolium) bromide), Glyphosate, AMPA (amino methyl phosphonic acid), DCF-DA (2-7-dichlorofluorescine diacetate), NAC (N-Acetyl cysteine) were purchased from Sigma-Aldrich.

#### 2.1.1 MTT

The solution was freshly prepared the day of the experiment by dissolving 5 mg/mL of powder in sterile Phosphate Buffered Saline (PBS—Sigma Aldrich).

#### 2.1.2 Glyphosate, AMPA and NAC

Stock solutions were freshly made the day of the experiments by dissolving the powder in serum-free cell culture medium. Then, stock solutions were diluted in complete cell culture medium to reach the working concentrations.

#### 2.1.3 DCF-DA

Stock solution was made by dissolving the powder in sterile dimethyl sulphoxide (DMSO—Sigma-Aldrich) and stored at −20°C, in the dark. Stock solution was diluted in sterile PBS with Ca^2+^/Mg^2+^ to reach the working concentration.

### 2.2 Cell culture

H9c2 cells (ATCC^®^ CRL-1446™) were purchased from Sigma-Aldrich. Cell culture was performed in Dulbecco’s Modified Eagle’s Medium (DMEM) with phenol red (Sigma-Aldrich) supplemented with 10% Fetal Bovine Serum (FBS - Sigma-Aldrich), 1% 200 mM L-Glutamine (Microgem), 1% penicillin/streptomycin (Sigma-Aldrich) at 37°C, 5% CO_2_, 25% O_2_. Cells were split at 80% confluence.

### 2.3 Cell viability

H9c2 cells were seeded in 96-well plates at 5 × 10^4^ cells/well and kept in incubator 24 h. Then, cells were starved O/N in DMEM 2% FBS and treated with different Gly or AMPA concentrations for different times. When necessary, cells were pretreated 1 h with NAC (100 µM). After the treatments, the medium was replaced, 10 µl MTT were added to each well and the plates were incubated 3 h at 37°C. Then, medium was discarded and the purple formazan crystals were dissolved in 100 µl DMSO. The optical density was measured in a microplate reader (Model 680—BioRad) at 570 nm. The experiment was performed on technical and biological triplicates.

### 2.4 Morphology

Cells were plated into Petri dishes and kept in complete medium for 24 h to allow cell adhesion. After the desired confluence (70%–90%) was reached, samples were treated with 10 or 20 mM of glyphosate or AMPA for 24 h (t24) or kept in culture medium. After the treatments, cells were washed with warm sterile PBS with Ca^2+^/Mg^2+^ and medium was replaced with a fresh one. All samples were observed under an optical microscope (Axiovert 200—Zeiss) at t0 or t24 with a 63X lens. Images were acquired through Infinity Analyze Software (Lumenera Corporation). At least five fields/sample have been analyzed. The experiment was performed on technical triplicates.

### 2.5 Transmission electron microscopy

H9c2 cells were plated into Petri dishes and kept in culture until reaching 80% confluence. Then, cells were treated with Gly 10 mM for 1 h or kept in culture medium (Control). Cells were gently washed with warm sterile PBS without Ca^2+^/Mg^2+^, detached with trypsin/EDTA 0.05%/0.02% (PAN Biotech), collected in tubes and centrifuged 5′ at 3000 rpm. Supernatant was discarded and pellet was fixed in 1% paraformaldehyde (Merck, Darmstadt, Germany), 1.25% glutaraldehyde (Fluka, St Louis, MO, United States) and 0.5% saccharose in 0.1 M Sörensen phosphate buffer (pH 7.2) for 2 h. For resin embedding, samples were post-fixed in 2% osmium tetroxide (SIC, Società Italiana Chimici) for 2 h and dehydrated in ethanol (Sigma Aldrich) from 30% to 100% (5 min each passage). After two passages of 7 min in propylene oxide, one passage of 1 h in a 1:1 mixture of propylene oxide (Sigma Aldrich) and Glauerts’ mixture of resins, samples were embedded in Glauerts’ mixture of resins (made of equal parts of Araldite M and the Araldite Harter, HY 964, Sigma Aldrich). In the resin mixture, 0.5% of the plasticizer dibutyl phthalate (Sigma Aldrich) was added. For the final step, 2% of accelerator 964 was added to the resin in order to promote resin polymerization at 60°C. Ultra-thin serial sections (70 nm thick) were cut using an Ultracut UCT ultramicrotome (Leica Microsystems, Wetzlar, Germany), stained with a solution of 4% UAR-EMS uranyl acetate replacement in distilled water and analysed using a JEM-1010 transmission electron microscope (JEOL, Tokyo, Japan) equipped with a Mega-View-III digital camera and a Soft-Imaging-System (SIS, Münster, Germany) for computerized acquisition of the images.

For mitochondria quantification, 4 ultra-thin sections 50 µm distant each other were considered for each experimental group with a magnification of 30000X. A total of 50 cells for experimental group were analysed and the number of impaired and unimpaired mitochondria was estimated in % based on their morphological features such as the shape of mitochondria, the morphology of the cristae and evidence of swelling.

### 2.6 ROS measurements

DCFH-DA is a non-fluorescent molecule permeable to cells. It is hydrolyzed at the intracellular level in dichlorofluorescine (DCFH), which is retained in the cell as it is no longer able to cross cell membranes. In the presence of H_2_O_2_, DCFH is oxidized forming the highly fluorescent DCF.

4 × 10^3^ cells/well were seeded in 96-well plates and kept in incubator O/N to allow adhesion. Cells were treated with different concentrations of Gly or AMPA for 1 or 2 h. After the treatments, cells were gently washed two times with warm PBS with Ca^2+^/Mg^2+^. 100 μl/well of 10 µM DCF-DA were added and the plates were incubated for 45 min at 37°C, covered by an aluminum foil. Cells were washed two times with warm PBS with Ca^2+^/Mg^2+^. The fluorescence intensity was measured at the wavelengths ex: 485 nm and em: 535 nm with a microplate reader (Infinite 200—Tecan). The experiment was performed on technical and biological triplicates. A control lane with only cells (NO DCF) was always included to subtract cellular auto-fluorescence.

### 2.7 Mitochondrial staining

MitoTracker Green FM™ (MTG—Thermo Fisher) is a fluorescent probe, which stains mitochondria independently from their metabolic activity.

5 × 10^3^ cells/well were plated in 24-well plates in complete DMEM and kept O/N in the incubator. Cells were washed with sterile warm PBS with Ca^2+^/Mg^2+^ and treated with different concentrations of Gly or AMPA for 2 or 24 h. After the treatments, cells were gently washed with sterile warm PBS with Ca^2+^/Mg^2+^. 100 nM MTG was added to each well and plates were incubated 30’ in the dark at 37°C. Samples were washed with warm sterile PBS with Ca^2+^/Mg^2+^ and observed under a fluorescence microscope (Axiovert 200—Zeiss) with a ×40 magnification lens. Images were acquired through Infinity Analyze Software (Lumenera Corporation) with a resolution of 480 × 360 pixels. At least five fields/sample have been analyzed. The experiment was performed on technical triplicates.

### 2.8 Statistical and computer analysis

Statistical analysis was performed using Graphpad Prism Software^®^ (version 9.00, GraphPad Software). Data are expressed as a mean ± SD. The differences between the groups were analyzed through statistical tests: ANOVA, one-way or two-way, or Kruskal-Wallis or Mann-Whitney *t*-test. Statistical significance has been set at *p* < 0.05.

## 3 Results

### 3.1 Effects of high doses of glyphosate and AMPA—Acute exposure

In order to evaluate whether an acute exposure to Gly or AMPA determines changes in cell viability in our cell model, we performed an MTT assay.

**FIGURE 1:**
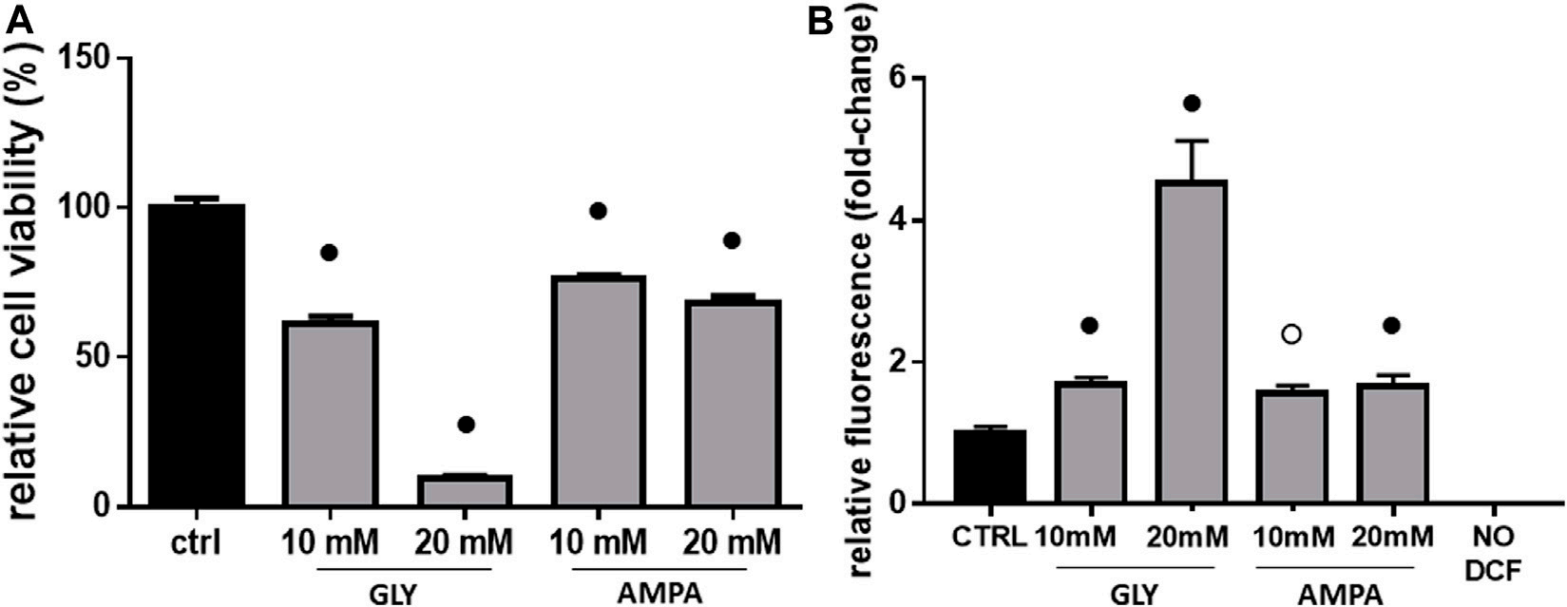
Cell viability & ROS production. The figure shows the relative histograms obtained from: **(A)** MTT and **(B)** DCF-DA assays. H9c2 cells were treated with 10 or 20 mM of glyphosate (GLY) or AMPA for 2 h. After the treatments, cell viability and ROS production were assessed, respectively, through MTT and DCF-DA assays. Mean ± SD; *p*-values: ○ < 0.0005; ● < 0.001 *vs*. control. NO DCF: control lane without the fluorescent probe.

After 2 h, Gly treatment diminished H9c2 viability in a concentration-dependent manner, with the most dramatic effect given by the highest dose. As shown in Figure 1, 10 and 20 mM treatment determined, respectively, 30% and 90% decrease in cell viability (Figure 1A). At equal doses, AMPA decreased cell viability from 20% to 30% (Figure 1A).

In light of the observed cytotoxic effects and considering that Gly, in the literature, is often associated with oxidative stress (Sardão et al., 2009; Kwiatkowska et al., 2014; Anifandis et al., 2017; Burchfield et al., 2019; Cao et al., 2021), we performed ROS measurements on H9c2 cells.

At 10 mM, there was a slight increase in ROS production compared to the control, without substantial differences between Gly and AMPA groups (Figure 1B). Treatment with 20 mM of Gly, instead, determined a 4-fold increase in ROS production (Figure 1B), which can explain the dramatic loss in cell viability (Figure 1A). This potent effect was not observed in 20 mM AMPA treated group, which ROS levels are comparable to 10 mM one (Figure 1B).

**FIGURE 2:**
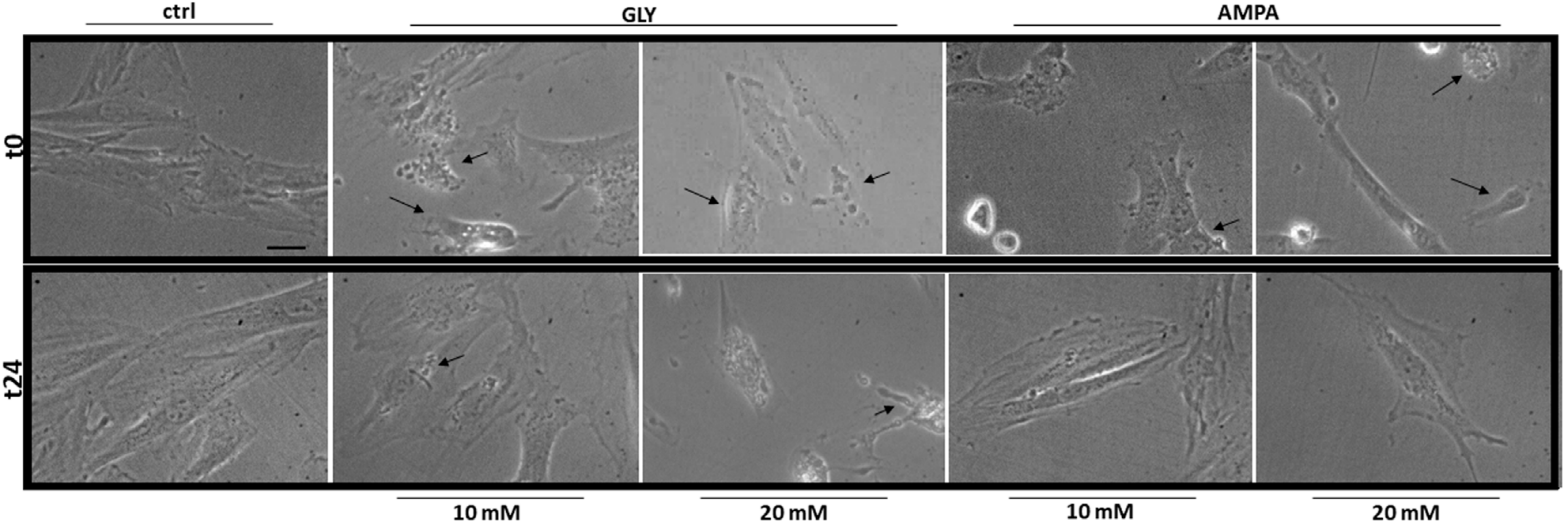
Morphology. The figure shows representative fields of H9c2 cells treated with 10 or 20 mM of glyphosate (GLY) or AMPA for 24 h. Images were acquired through a camera connected to an inverted microscope at the start (t0—top panels) and at the end (t24—bottom panels) of the treatments with a 63X lens (scale bar = 10 µm).

After 24 h (t24), signs of membrane blebbing and cell shrinkage are still present in Gly-treated group (Figure 2, bottom panels); many rounded and floating cells were clearly visible in the plates at 20 mM, together with strong signs of cytoplasmic cavitation. The same morphological alterations were not observed in AMPA treated group (Figure 2, bottom panels).

After analysis of phenotypical changes (Figures 1, 2), additional MTT and DCF-DA assays were performed in a shorter time-range, focusing on 10 mM Gly treatment, which effects were not too deleterious on the selected cell model.

**FIGURE 3:**
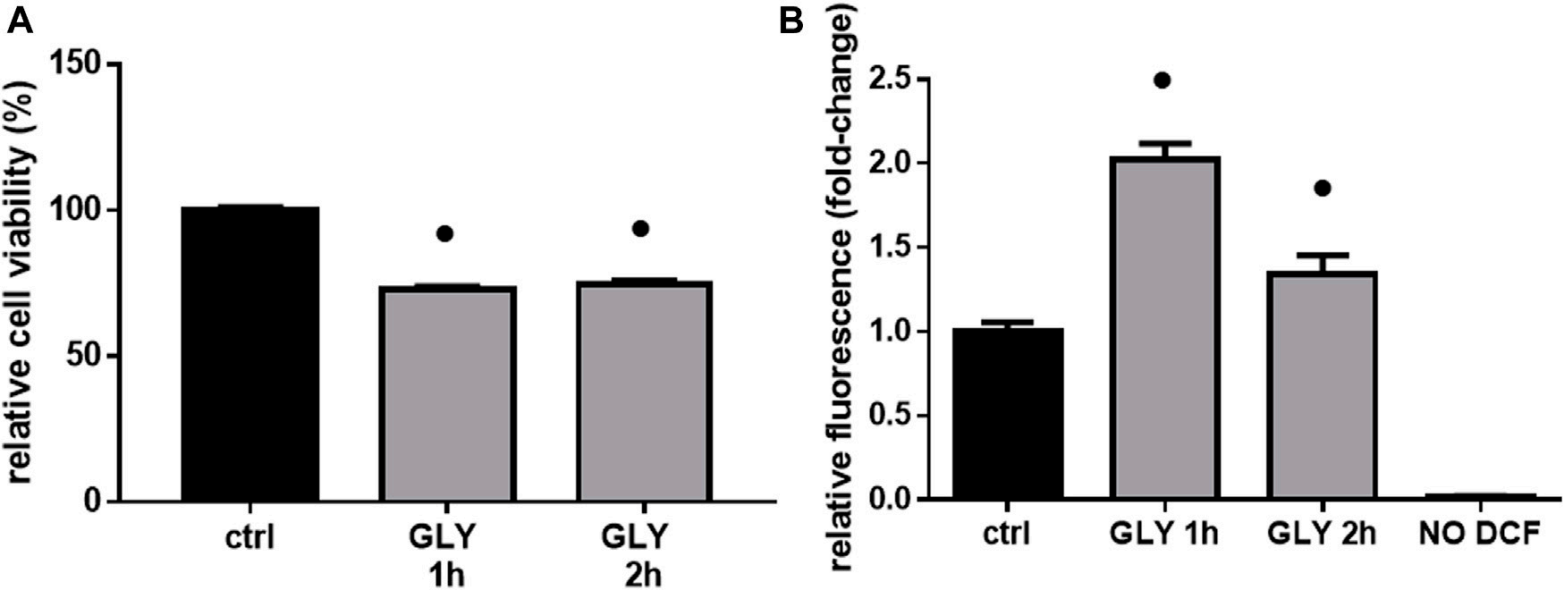
Cell viability & ROS production. The figure shows the relative histograms obtained from: **(A)** MTT and **(B)** DCF-DA assays. H9c2 cells were treated with 10 mM of glyphosate (GLY) for 1 or 2 h. After the treatments, cell viability and ROS production were assessed, respectively, through MTT and DCF-DA assays. Mean ± SD; *p*-values: ● < 0.0001 vs. control. NO DCF: control lane without the fluorescent probe.

Interestingly, cell viability did not change when comparing 1 h and 2 hours-treatment groups (Figure 3A), while ROS production was significantly higher after 1 h (Figure 3B), confirming an early response of H9c2 cells to these levels of Gly exposure.

**FIGURE 4:**
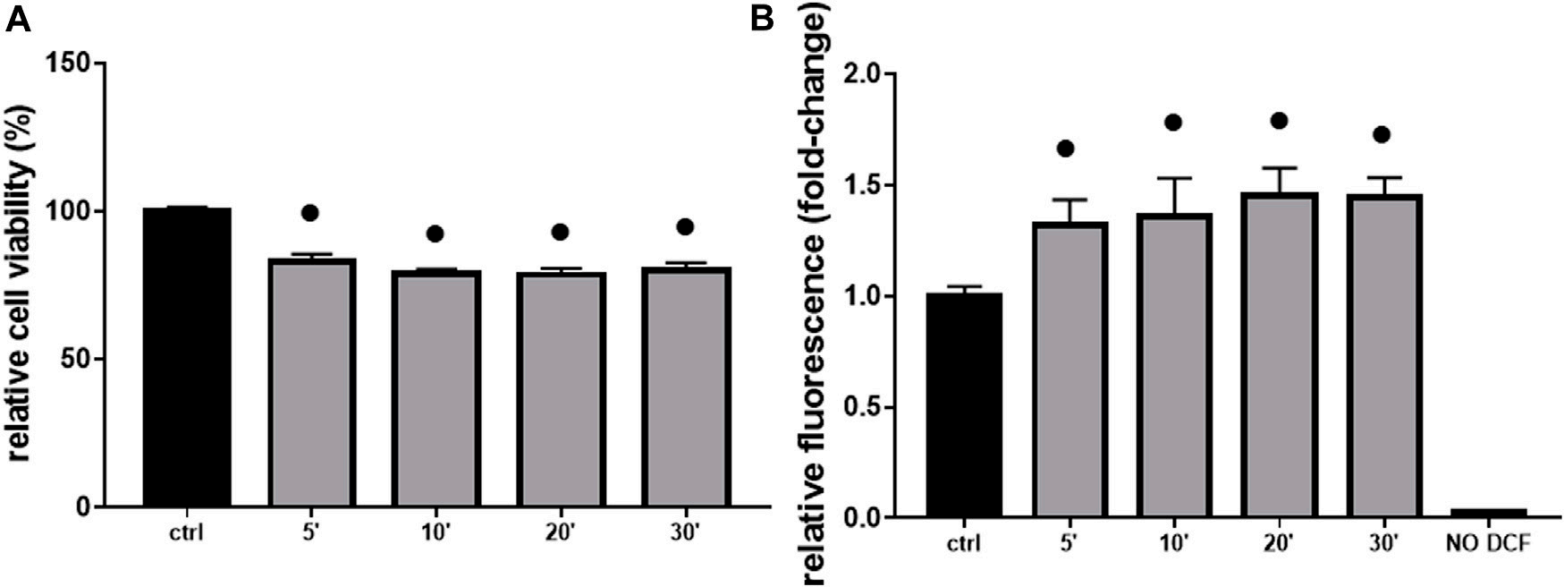
Cell viability & ROS production. The figure shows the relative histograms obtained from: **(A)** MTT and **(B)** DCF-DA assays. H9c2 cells were treated with 10 mM of glyphosate at different time points (from 5 to 30 min). After the treatments, cell viability and ROS production were assessed, respectively, through MTT and DCF-DA assays. Mean ± SD; *p*-values: ● < 0.0001 *vs*. control. NO DCF: control lane without the fluorescent probe.

As additional confirmation, both a decrease in cell viability (≡ 20%, Figure 4A) and an increase in ROS production (≡ 1.5 fold-change, Figure 4B) were significant in H9c2 cells already after 5 min of 10 mM Gly. However, the most appreciable effect was reached after 1 h (Figures 3, 4).

**FIGURE 5:**
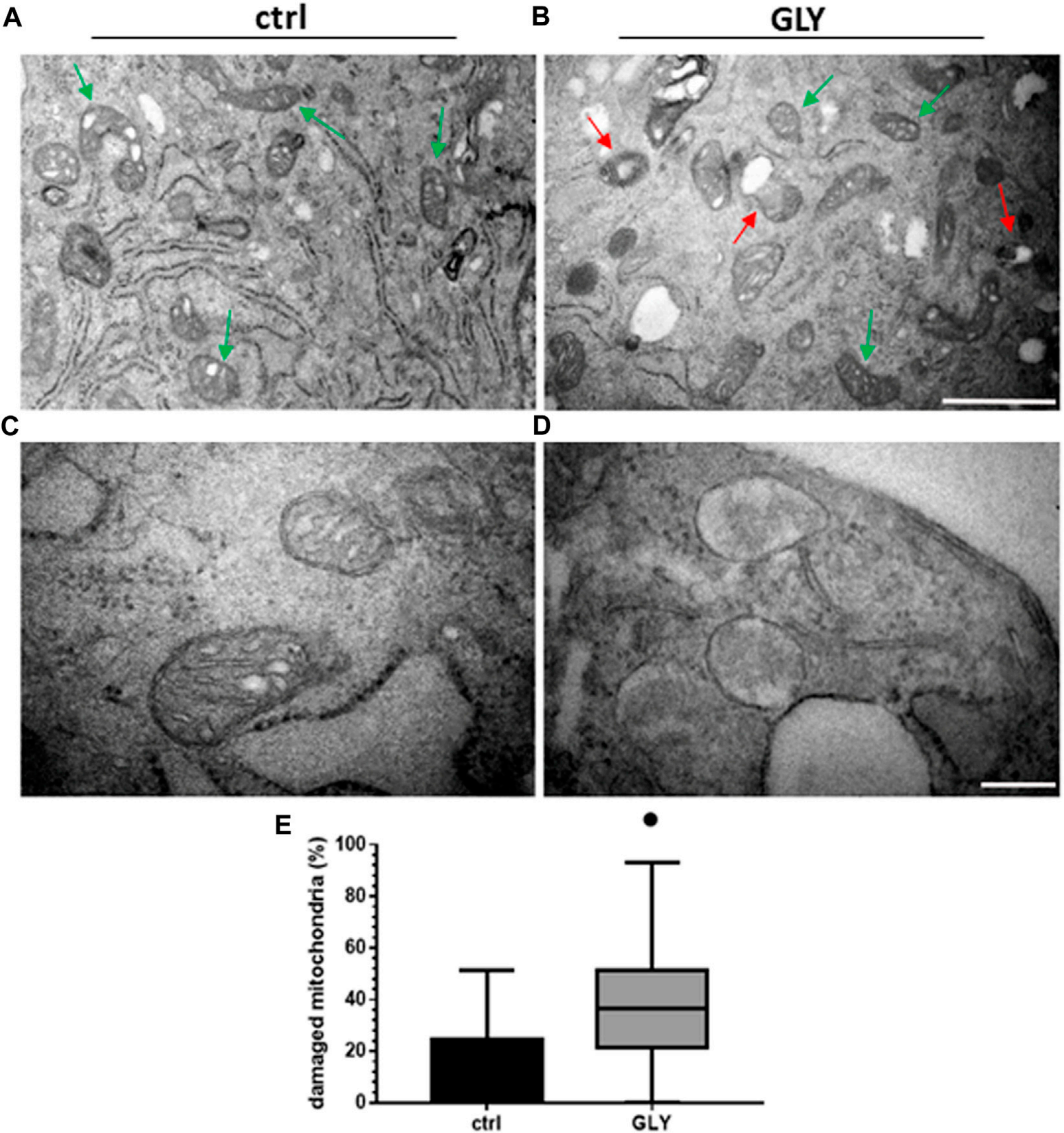
Ultrastructural analysis and mitochondria quantification. The figure shows: **(A)** Ultrastructure of HM in H9c2 cells control group (green arrows); **(B)** morphology of DM (red arrows) and HM (green arrows) in GLY group (10 mM, 1 h of treatment) (Scale bar = 1 µm); **(C)** higher magnification of HM in control group and **(D)** higher magnification of DM in GLY group (Scale bar = 0.2 µm). In (**E)** the relative boxplots of the mitochondrial count are shown. *p*-values: ● < 0.0001 *vs*. control.

Given the significant and rapid production of ROS, an involvement of Gly-driven mitochondrial functional impairment was postulated. Therefore, H9c2 cells were treated with Gly 10 mM for 1 h and analyzed using transmission electron microscopy. The morphology of mitochondria was further investigated by transmission electron microcopy that allowed to access healthy mitochondria with intact double membrane structure, cristae and cristae space easily detectable in the control group (Figures 5A, C); several swollen mitochondria without cristae, instead, are detected in Gly-treated group (Figures 5B, D). Furthermore, the number of perinuclear mitochondria was quantified. We determined two populations: 1) “*healthy mitochondria*” (HM) showing normal morphology, *cristae* structure and intact membrane; 2) “*damaged mitochondria*” (DM) with degenerated or swollen *cristae*. To calculate the percentage of DM, we used the formula:

$$\%\;of\;DM=\frac{DM}{\left(HM+DM\right)}*100$$

As shown in Figure 5E, the percentage of damaged mitochondria was significantly higher in Gly-treated group compared to control, potentially explaining the observed cytotoxic effects.

### 3.2 Effects of medium-to-low doses of glyphosate and AMPA—Acute exposure

**FIGURE 6:**
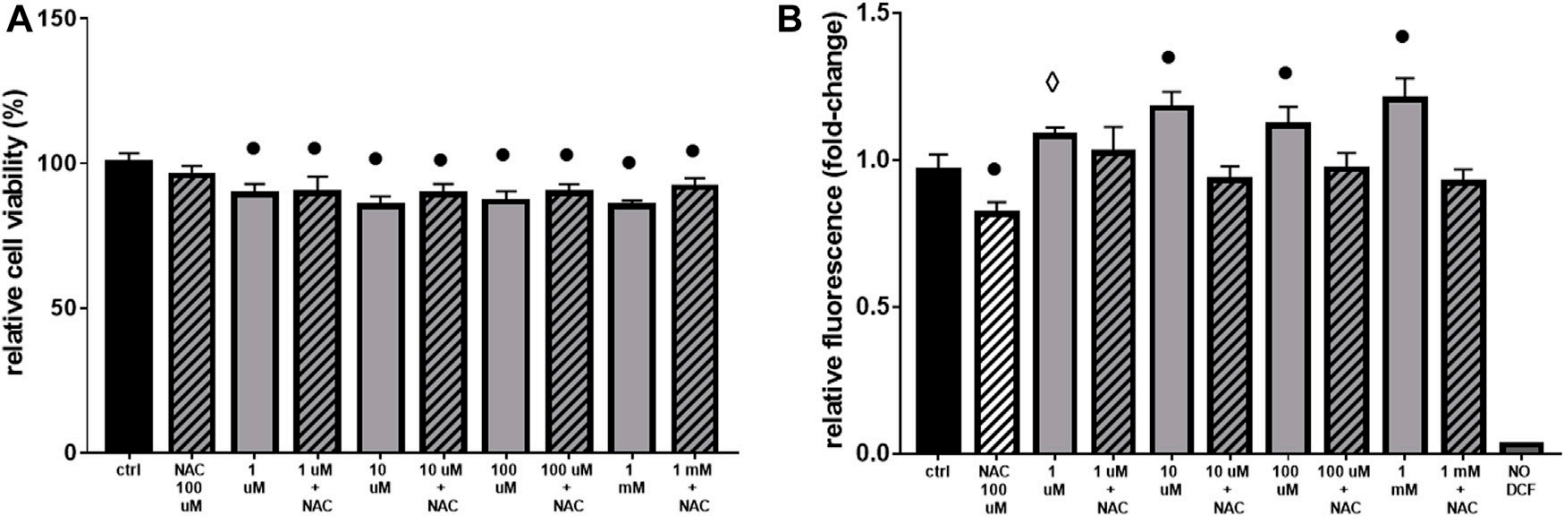
Cell viability and ROS production. The figure shows the histograms obtained from: **(A)** MTT and **(B)** DCF-DA assays. H9c2 cells were treated with 1 μM to 1 mM of glyphosate and/or 100 µM of NAC for 2 h. After the treatments, cell viability and ROS production were assessed, respectively, through MTT and DCF-DA assays. Mean ± SD; *p*-values: ⋄ < 0.005; ○ < 0.0005; ● < 0.0001 vs. control. NO DCF: control lane without the fluorescent probe.

The acute exposure of H9c2 cells from medium (1 mM) to very low (1 µM) doses of Gly, produced similar effects seen before (Figure 1), although to a lesser extent. In terms of cell viability and ROS production, the treatments determined, respectively, a decrease from 10% to 15% (Figure 6A) and an increase from 1.1 to 1.2 fold-change (Figure 6B).

The use of the antioxidant NAC, even if effective in lowering ROS production (Figure 6B), was not able to totally restore cell viability (Figure 6A).

After the observation of Gly- and AMPA-induced production of reactive oxygen species, we wanted to test if there were variations in mitochondrial mass and distribution. To do so, we probed mitochondria with the fluorescent molecule MitoTracker Green FM™ after 2 or 24 h of Gly or AMPA treatment.

**FIGURE 7:**
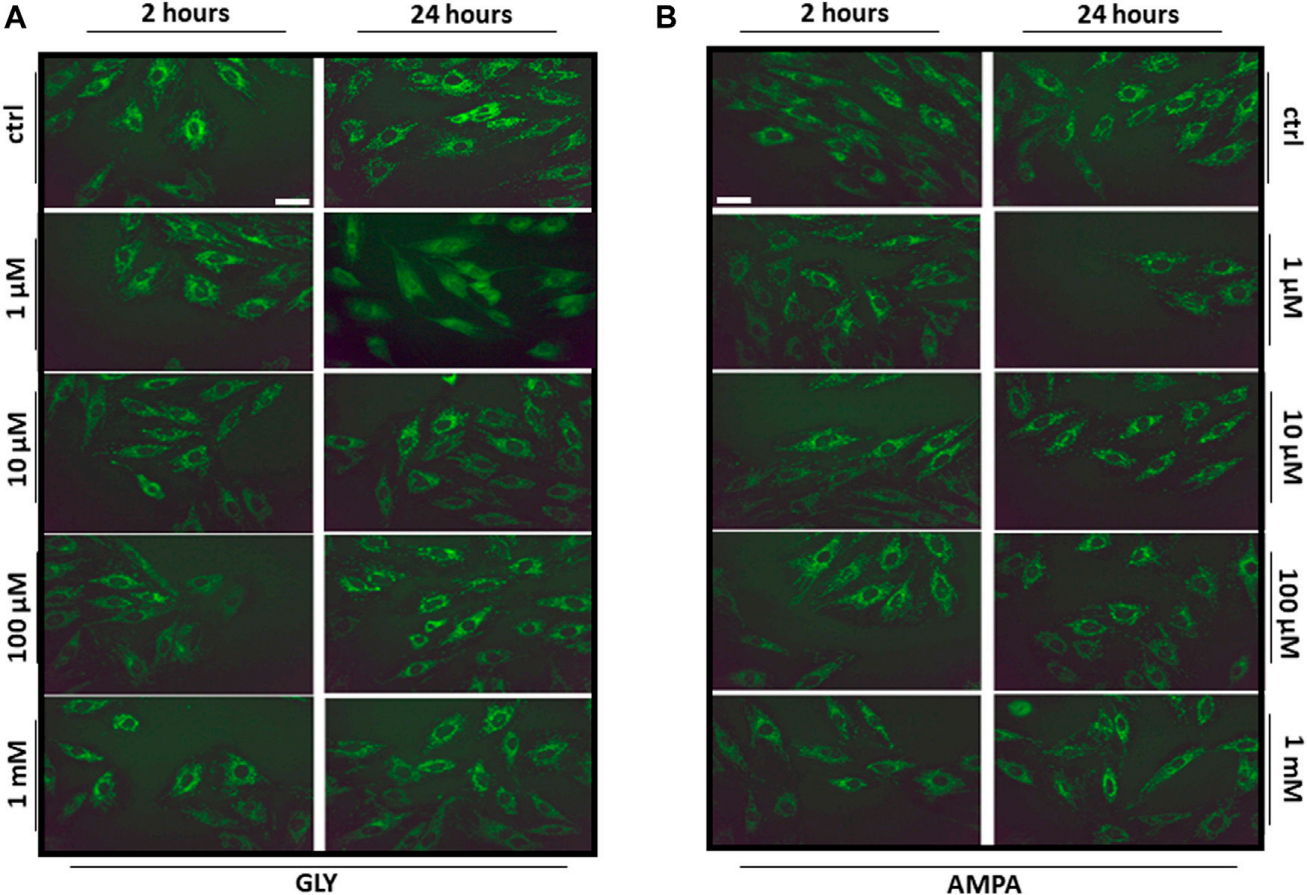
Mitochondrial distribution. The figure shows the representative images (40X, scale bar = 10 µm) of H9c2 cells stained with the mitochondrial fluorescent dye MitoTracker Green FM. H9c2 cells were treated with 100 nM to 1 mM of **(A)** glyphosate (GLY) or **(B)** AMPA for 2 or 24 h.

Mitochondrial distribution, as shown in Figures 7A, B, appeared homogeneous and no variations in fluorescence intensity were detected, suggesting that both mitochondrial dynamics and mass were preserved. However, we cannot totally exclude that the inability to find any relevant change, could be associated with a limitation of the technique used, which has, indeed, a limited resolution. Moreover, the probe stains all mitochondria independently from their activity, so it was no possible to distinguish healthy and damaged populations.

### 3.3 Effects of medium-to-low doses of glyphosate and AMPA—Sub-chronic exposure

Given the scarce effects of Gly on ROS production (Figures 6A, B) and since there were not changes in mitochondrial distribution and mass after 24 h of Gly (Figure 7A) or AMPA (Figure 7B) exposure, we hypothesized that H9c2 cells were able to overcome the injury. To verify this hypothesis, we tested cell viability of H9c2 cells after prolonged exposure (24 and 48 h) to low doses (1 μM–1 mM) of Gly or AMPA.

**FIGURE 8:**
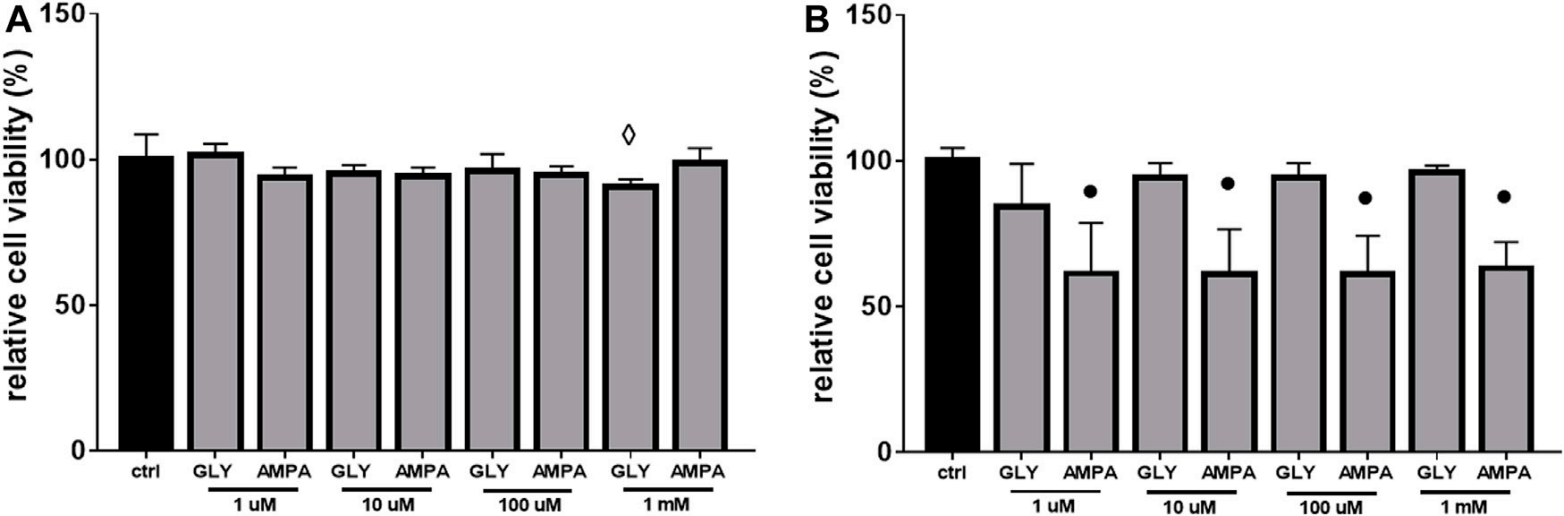
Cell viability. The figure shows the relative histograms obtained from MTT assays. H9c2 cells were treated with 1 μM to 1 mM of glyphosate (GLY) or AMPA for **(A)** 24 h or **(B)** 48 h. Mean ± SD; *p*-values: ⋄ < 0.005; ● < 0.0001 *vs*. control.

As expected, after 24 h of low doses of Gly exposure, cell viability is totally rescued, except for the 1 mM dose (Figure 8A). After 48 h, the control phenotype was restored under all doses (Figure 8B).

As regards to AMPA treated-group, after 24 h, cell viability was comparable to control cells (Figure 8A). Surprisingly, after 48 h of exposure, cell viability decreased by ≡ 40% at all doses (Figure 8B).

## 4 Discussion

Gly is considered an environmental pollutant as active compound of a large part of non-selective herbicidal largely used worldwide in the last 50 years (Torretta et al., 2018). As a matter of fact, traces of Gly and AMPA (its main degradation product) are commonly detected in samples of water, soil and food (Bai and Ogbourne, 2016; Bonansea et al., 2017; Silva et al., 2018; Xu et al., 2019). This diffuse contamination leads to a constant exposure, representing both an ecological and a health concern for humans and animals. Despite its plant-specific mechanism of action, Gly has been proven to have either acute or chronic toxicity in different animal species, including mammals.

### 4.1 Glyphosate effects

At high doses, Gly treatment determines a great reduction in myoblasts viability after 2 h (Figure 1A). The response appears very early, since 10 mM treatment is able to reduce cell viability already after 5 min (Figure 4A). Furthermore, cell shrinkage and membrane blebbing are already visible soon after the application of the treatments (Figure 2, top panels). Signs of cell damage are still present after 24 h (Figure 2, bottom panels). Coupled to the reduction in cell viability, these morphologic alterations suggest an involvement of apoptotic pathways (Sardão et al., 2009; Gui et al., 2012; Zhang et al., 2018; Noritake et al., 2020). Benachour and Séralini (2009) showed that *in vitro* pure Gly treatment caused apoptosis via caspase (cas)-3 and -7 activation, already after 6 h, in three different human cell lines. Gly-dependent increase in cas-3, -8 and -9 activity was also recently confirmed in human peripheral blood mononuclear cells (hPBMCs) (Kwiatkowska et al., 2020). Moreover, in a neuroblastoma cell line (SHSY-5Y), 5 mM Gly treatment altered the expression of different apoptosis-related genes such as BAX, BCL2, CASP3 and CASP9 (Martínez et al., 2020).

The toxic effects we observed were related, at least in part, to ROS production and mitochondrial abnormalities. Mitochondria are, in fact, key players in maintaining cellular redox status and homeostasis. Upon a toxic stimulus, mitochondria may trigger an apoptotic response through cytochrome c release followed by the activation of cas-9-dependent pathway (Orrenius, 2004). A dose of 10 mM Gly determined a great production of ROS already after 5 min (Figure 4B), reaching the peak after 1 h (Figure 3B). In addition, 1 h of Gly treatment rapidly provoked mitochondrial disruption (Figures 5A, B). This is in line with what shown in hPBMCs, in which 4 h *in vitro* Gly treatment, from 0.05 mM, caused a significant reduction in mitochondrial membrane potential (ΔѰm) and a consistent ROS production. These effects were markedly increased at 5 mM concentration (Kwiatkowska et al., 2020). H9c2 viability after 1 or 2 h of Gly exposure was comparable (Figure 3A), altogether suggesting that the damage could occur during the first hour. However, it remains a speculation since we did not checked these data in a longest time-window for this range of Gly concentration.

The same drastic effects were not detected at lower concentrations (1 μM–1 mM), in which there was only a slight (although significant) variation in cell viability (Figure 6A) and ROS production (Figure 6B) after acute treatment. Similar results were obtained from Kim et al. (2013): the researchers found that the treatment with pure Gly up to 10 µM was not able not alter H9c2 features in terms of caspases activation, cell morphology and ΔѰm. As a further confirmation of the low toxicity, the sub-chronic exposure (24 or 48 h) of H9c2 to low doses of Gly showed a total rescue of the phenotype in terms of cell viability (Figures 8A, B) and no variations in mitochondrial dynamics (Figure 7A) or cell morphology (data not shown), suggesting that the cells were able to recover from the damage. A similar type of behaviour has been already reported by Townsend et al. (2017), which demonstrated that Gly is lethal to Raji cells (a line of lymphoblast-like cells) at concentrations above 10 mM, while no cytotoxic effects were observable at concentrations at or below 100 μM. Furthermore, in their study, acute (from 30 to 60 min) Gly treatment in concentrations between 1 and 5 mM induced significant DNA damage, which was totally recovered after 2 h.

Overall, our original results are not in contrast with what previously reported in literature. Gly appears toxic, on average, at- or above 1 mM in different mammalian and non-mammalian cell types, while at low doses it is relatively safe. The toxicity mechanisms seem to be related to oxidative stress, induced by mitochondrial dysfunctions or disruption of antioxidant systems (Contardo-Jara et al., 2009; Kwiatkowska et al., 2014; Lopes et al., 2014; Jin et al., 2018; Vanlaeys et al., 2018; Martínez et al., 2020; Nerozzi et al., 2020; Madani and Carpenter, 2022; Strilbyska et al., 2022).

It remains unclear whether Gly exerts its toxicity by acting in an intra- or extra-cellular manner. Unfortunately, as of today, it is not known whether glyphosate is transported into mammalian cells and how it may vary across different cell lines. A 2016 study performed on a human epithelial cell line suggests an active uptake mediated by the L-type aminoacid transporter (LAT) (Xu et al., 2016). We evaluated whether our cells could use this carrier for Gly uptake. To do so, we co-treated the cells with different doses of glyphosate (5, 10 and 20 mM) and a specific LAT-1 inhibitor (2-aminobicyclo-(2,2,1)-heptane-2-carboxylic acid, BCH) in acute settings (1 and 2 h). We, then, assessed cell viability and ROS production through MTT and DCF-DA assays, respectively, that did not show any changes in Gly-driven cytotoxicity (data not shown), suggesting that cardiac myoblasts use a different type of transport system and/or that Gly toxicity relies on a receptor-mediated signalling.

### 4.2 AMPA effects

Cells exposure to AMPA showed two types of responses. There was an acute cytotoxic response to high doses (10 or 20 mM), as demonstrated by a reduction in cell viability (Figure 1A) and an increase in ROS production (Figure 1B). Membrane blebbing, cell shrinkage and cytoplasmic cavitation were observable at t0 (Figure 2, top panels), but not after 24 h of treatment (Figure 2, bottom panels). Overall, in this range of concentrations, AMPA treatment was less toxic than Gly. Kwiatkowska et al. (2020) observed an analogous behavior: in hPBMCs, the treatment with AMPA induced hydroxyl radical formation only at the highest concentration (5 mM), while Gly treatment was effective already at 0.05 mM. Similarly, in a study from 2018, it was observed an increase in ROS levels in hPBMCs exposed to 1 mM Gly, but not to the same concentration of AMPA (Woźniak et al., 2018). In SHSY-5Y cells, after 48 h of exposure to 10 mM AMPA there was a significant increase is ROS production, while Gly exerted the same effect at 5 mM (Martínez et al., 2020).

Conversely, when treated sub-chronically at low doses (from 1 to 1 mM), H9c2 cells showed a late cytotoxic response to AMPA. After 48 h, there was a decrease in cell viability about 40% at all doses (Figure 8B). This was somehow unexpected, given the scarce amount of data about AMPA effects (especially in mammals) (Grandcoin et al., 2017; Bailey et al., 2018; Stur et al., 2019), and represents a result that need to be explored with more detail. A non-monotonic response to sub-lethal doses of AMPA was recently reported in amphibians. In such experimental model, the chronic treatment with low (0.07 μg/L) and medium (0.32 μg/L) doses of AMPA determined a significant dysfunction of the antioxidant machinery, which authors suggest to be linked to a hyper-stimulation of catalase activity, while high doses (3.57 μg/L) did not recapitulate the same effect (Cheron et al., 2022). We hypothesize that the early response could be due to a direct extracellular damage (as the binding with a receptor), while the late one could be secondary to bioaccumulation. Accordingly, it was demonstrated that, in hPBMCs, AMPA treatment determined an increase in both cas-8 [generally associated with the death receptors-mediated apoptotic pathway (Orrenius, 2004)] and cas-9 [involved in the mitochondrial-mediated apoptotic pathway (Orrenius, 2004)] activity (Kwiatkowska et al., 2020), supporting the hypothesis that the molecule is able to trigger both types of response. The activation of cas-3 and cas-9 pathways, following 48 h of AMPA treatment, was also reported by Martínez et al. (2020) in SHSY-5Y cells.

The fact that Gly treatment did not determine the same effects, could have two means: (I) Gly is not actively metabolized in AMPA neither inside nor outside our cells; (II) the kinetic of Gly to AMPA biotransformation is very slow, so more time is necessary to start to see the effects (Bailey et al., 2018).

### 4.3 Conclusion

Overall, we confirmed in our model previous *in vitro* studies indicating that pure Gly is toxic when administered at high concentrations, causing alterations in cell viability, morphology and mitochondrial health. At low doses, Gly causes only a slight cytotoxic response and the phenotype is rescued within 24 h. AMPA recreates almost the same effects, but with a lesser extent. Moreover, we provided new evidences about a late cytotoxic response to low doses of sub-chronically administered AMPA. In each condition, mitochondria and the antioxidant machinery are likely to be key mediators, a finding which is largely supported by the literature. Unfortunately, the comprehension of the mechanisms by which Gly is possibly imported into mammalian cells is very limited, nor is clear if it is actively or inactively metabolized within the cells. Unveiling these aspects would help to clarify whether the damage is receptor-mediated or if it occurs after the internalization of the molecules. Furthermore, it is of pivotal importance to have a reliable measure of the real human exposure to glyphosate and AMPA, in order to critically evaluate all the scientific data obtained as of today. Since the main route of exposure of the general population to Gly is through the diet, it is pivotal to perform quality control of the agro-food chain. In particular, on those foods which are more likely to contain Gly such as fish/meat and derivatives, cereals and derivatives, honey and beverages such as tea, beer and wine. Some studies have been already conducted and are reported in a recent review by Soares et al. (2021). To do so, there is the urge to develop standardized quantification systems with good sensitivity (that should be well below the maximum residue limit established) but also affordable in terms of technical equipment and costs, a goal achievable with HPLC-related methodologies. Last, in order to shed light on the debate about Gly safety, it would be helpful to distinguish between the damages directly related to the pure molecules and its metabolites and the ones mediated (or amplified) by the adjuvants, i.e., the surfactants, present in the different GBH formulations.

This study needs further research to address additional scientific concerns: first, we did not included AMPA in all of the experiments, since we did not expect to observe any appreciable effect (especially at low doses); second, we did not examine in depth the effects of the chronic exposure of the two substances. However, the evaluation of a chronic treatment in an *in vitro* environment is limited and this study was intended as a pilot to identify a sub-toxic range, coherent with the environmental exposure, to evaluate the chronic toxicity of Gly *in vivo*.
